# Continuity of care in diverse ethnic groups: a general practice record study in England

**DOI:** 10.3399/BJGP.2022.0271

**Published:** 2022-11-01

**Authors:** Mai Stafford, Laia Bécares, Brenda Hayanga, Mark Ashworth, Rebecca Fisher

**Affiliations:** The Health Foundation, London.; School of Population Health and Environmental Sciences, King’s College London, London.; School of Population Health and Environmental Sciences, King’s College London, London.; School of Population Health and Environmental Sciences, King’s College London, London.; The Health Foundation, London.

**Keywords:** continuity of care, ethnicity, general practice, inequalities, multiple long-term conditions

## Abstract

**Background:**

GPs and patients value continuity of care. Ethnic differences in continuity could contribute to inequalities in experience and outcomes.

**Aim:**

To describe relational continuity of care in general practice by ethnicity and long-term conditions.

**Design and setting:**

In total, 381 474 patients in England were included from a random sample from the Clinical Practice Research Datalink (January 2016 to December 2019).

**Method:**

Face-to-face, telephone, and online consultations with a GP were included. Continuity, measured by the Usual Provider of Care and Bice–Boxerman indices, was calculated for patients with ≥3 consultations. Ethnicity was taken from the GP record or linked Hospital Episode Statistics data, and long-term conditions were counted at baseline. Multilevel regression models were used to describe continuity by ethnicity sequentially adjusted for: a) the number of consultations, follow-up time, age, sex, and practice-level random intercept; b) socioeconomic deprivation in the patient’s residential area; and c) long-term conditions.

**Results:**

On full adjustment, 5 of 10 ethnic minority groups (Bangladeshi, Pakistani, Black African, Black Caribbean, and any other Black background) had lower continuity of care compared with White patients. Continuity was lower for patients in more deprived areas and younger patients but this did not account for ethnic differences in continuity. Differences by ethnicity were also seen in patients with ≥2 long-term conditions.

**Conclusion:**

Ethnic minority identity and socioeconomic deprivation have additive associations with lower continuity of care. Structural factors affecting demand for, and supply of, GPs should be assessed for their contribution to ethnic inequalities in relational continuity and other care quality domains.

## INTRODUCTION

Relational continuity of care — understood to mean an ongoing therapeutic relationship between patient and practitioner — is associated with a range of positive outcomes. These include lower mortality, fewer hospital admissions, fewer condition-related complications, and higher patient satisfaction.^1^^–^^4^ Relational continuity of care may be particularly important for people with multiple long-term conditions^5^ as it has also been associated with lower risk of unscheduled hospital care and slower progression to additional conditions.^6^^,^^7^ UK evidence on the association between number of long-term conditions and relational continuity is mixed. One national study found that having more long-term conditions was associated with lower continuity.^8^ This was primarily driven by their higher number of consultations, as continuity of care indices tend to be negatively correlated with total number of consultations.^3^^,^^9^ One study set in London found no association between number of long-term conditions and continuity.^10^

Evidence indicates a higher prevalence of multiple long-term conditions in people from ethnic minority backgrounds in the UK.^11^ Among people with multiple long-term conditions, there is evidence of poorer outcomes including higher mortality and more emergency admissions to hospital for some ethnic minority groups compared with the White majority.^11^^–^^13^ One possibility is that ethnic inequalities in health care, such as continuity of care, contribute to these poorer outcomes.

Few UK studies describe continuity of care by ethnicity. Data from the General Practice Patient Survey (GPPS) show a decline in relational continuity between 2011 and 2017 across most sociodemographic groups^14^^,^^15^ but with a greater decline for ethnic minority groups than for patients of White ethnicity.^16^ The GPPS also shows that patients from some ethnic minority groups have a higher preference to see a particular GP but less success in doing so.^17^ The association between ethnicity and poorer experience of continuity with GPs has persisted over time,^18^^,^^19^ and has been found in a recent study triangulating survey data and GP records.^20^ These studies have not focused on possible ethnic inequalities in care for people with multiple long-term conditions.

The aims of the present study were to describe relational continuity of care in general practice by ethnicity at differing levels and types of long-term conditions. Although there are other dimensions of continuity of care including management continuity, relating to whether care is managed in a consistent and coordinated way, and informational continuity, relating to how the information held about a patient is shared and draws on their preferences and health goals, this study focused on relational continuity. Both the total count of conditions and the combinations of mental and physical conditions were considered. Mental health conditions are common among people with multiple conditions, and those with mental–physical multimorbidity are at greater risk of poor outcomes than those with only physical or only mental health conditions.^21^^–^^24^ They may particularly benefit from relational continuity for support to manage their complex care needs.^25^ Existing evidence suggests that mental–physical multimorbidity is less prevalent in the healthcare records of some ethnic minority groups compared with patients of White ethnicity.^26^^,^^27^ In the current study, the hypotheses were:

- that people from ethnic minority backgrounds would have lower continuity than those of White ethnicity;
- that these patterns would be seen for people with multiple physical health conditions and those with a combination of physical and mental health conditions; and
- given the greater levels of socioeconomic deprivation experienced by many ethnic minority groups, and the negative association between deprivation and continuity,^16^ that area deprivation would be on the explanatory pathway — so adjustment for deprivation would partly explain ethnic inequalities in continuity of care.

**Table:** How this fits in

Nationally representative survey data show lower continuity of care for most ethnic minority groups. To the authors’ knowledge, this is the first national study to examine ethnic inequalities in continuity of care using GP records. The study found that relational continuity of care was lower for people from Black African, Black Caribbean, any other Black background, Bangladeshi, and Pakistani ethnic groups. These ethnic inequalities are not accounted for by socioeconomic deprivation and are seen for people with and without multiple long-term conditions.

## METHOD

### Participants

A random sample of 690 000 patients was drawn from the Clinical Practice Research Datalink (CPRD Aurum^28^). CPRD includes pseudonymised primary care records for over 40 million patients. Patients eligible for the present study met the following criteria on 1 January 2016: registered in a CPRD practice; aged ≥18 years; eligible for linkage to Hospital Episode Statistics (Admitted Patient Care) and Office for National Statistics area deprivation data (2015 Index of Multiple Deprivation based on patient’s residential Lower Layer Super Output Area); and with acceptable data quality.

All consultations with a GP (including locums and sessional GPs) that took place between 1 January 2016 and 31 December 2019 and were face-to-face, telephone, or online were included.^29^

### Measures

Continuity of care was captured on two widely used indices. The two indices are positively correlated but are differently affected by the total number of GPs that a patient sees.

The Usual Provider of Care (UPC) index captures the concentration of care with a specified GP.^30^ It is calculated as the maximum number of visits to the same GP divided by the total number of visits over a specified time period. Although the UPC is straightforward to interpret, it does not account for the fact that a patient may have high relational continuity with >1 GP. For example, a patient with five visits to the same GP plus five visits to five different GPs would have the same UPC (0.5) as a patient with 10 visits spread equally across only two GPs.

The Bice–Boxerman Continuity of Care (COC) index captures the concentration of visits across all the GPs seen.^31^ It is calculated as the square of the number of visits with a GP, summed across all GPs and divided by the product of the total number of visits and the total number of visits minus 1. Here, the COC index would be 0.22 and 0.44, respectively, for the two scenarios described above. Patients with a minimum of three consultations during follow-up from 2016 to 2019 were included in the main analysis. Shorter follow-up was also considered in sensitivity analysis.

Ethnicity was taken from SNOMED CT codes in the primary care record or, if missing, from the Hospital Episode Statistics (Admitted Patient Care) record, using a previously published algorithm for dealing with multiple observations of ethnicity.^32^ The England and Wales 2011 Census categories were used but the White British and other White ethnic groups were combined to reduce the number of missing values in the main analysis. In sensitivity analysis, the White ethnicity group and the mixed ethnicity group were disaggregated.

The number of long-term conditions was counted on 1 January 2016 from a list adapted from previous analysis of CPRD^33^ and Scottish primary care data^34^ (Supplementary Table S1) based on published SNOMED CT code lists.^35^ In addition to the total count, patients with four different combinations of conditions were identified: zero or one condition; two or more physical health conditions; two or more mental health conditions; and one or more physical and one or more mental health condition.

### Statistical analysis

All analyses were adjusted for number of consultations and for length of follow-up to account for some patients leaving the practice or dying before 31 December 2019, or the practice ceasing contributions to CPRD. Using multilevel linear regression models to allow for the clustering of patients within the same GP practice, first bivariate associations between each covariate and the two continuity of care indices were estimated. Then, estimated differences in continuity of care across ethnic minority groups were compared with the majority White ethnic group with adjustment for: a) age and sex; b) Index of Multiple Deprivation based on the residential postcode of the patient; and c) number or combination of long-term conditions.

In sensitivity analysis, continuity of care over a period of 12 months was calculated with the sample limited to patients with at least three consultations between 1 January 2016 and 31 December 2016. More detailed ethnic groups were also examined for the subset of patients where this was available, breaking down the White ethnic group as British/Irish/other White, and the mixed ethnic group as White, Asian/White, African/White, and Caribbean/other mixed.

## RESULTS

**Figure 1.**
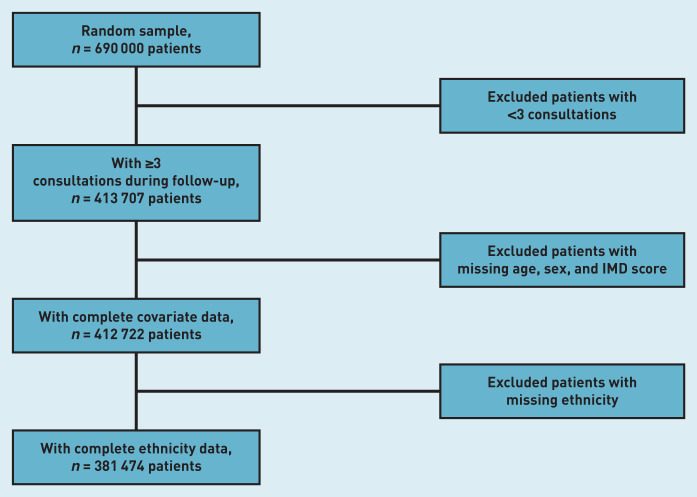
*Analytical sample flow chart. IMD = Index of Multiple Deprivation.*

From the initial sample, patients with <3 GP consultations during follow-up were excluded (Figure 1). Males and younger patients were overrepresented in those excluded. In total, a further 32 233 patients with missing data were excluded. Males, younger patients, and those living in the least deprived areas were overrepresented in those further excluded because of missing ethnicity data (Supplementary Table S2).

Females and patients living in the least deprived fifth of areas were overrepresented in the analytical sample (Table 1). Over a maximum of 4 years of follow-up, continuity on the UPC index ranged from 0.42 for those aged 18–29 years to 0.48 for those aged 70–79 years, and the COC index ranged from 0.21 to 0.30 in the same age groups. One-third of patients had ≥2 long-term conditions. This ranged from under 15% in people of Chinese ethnicity to over 35% in people of White ethnicity (Supplementary Table S3). The UPC index was lowest for people of Bangladeshi ethnicity (0.41) and was 0.45 for people of White ethnicity (Table 1). This difference translates to one additional consultation with the most frequently seen GP for every 25 visits for the White ethnic group compared with the Bangladeshi ethnic group.

**Table 1.** Continuity of care by demographic characteristics

**Characteristic**	**Total patients, *n* (%)**	**UPC, mean (SD)**	**COC, mean (SD)**
**Age group, years**			
18–29	58 920 (15.4)	0.42 (0.20)	0.21 (0.21)
30–39	64 348 (16.9)	0.42 (0.20)	0.22 (0.21)
40–49	62 563 (16.4)	0.44 (0.20)	0.24 (0.22)
50–59	66 207 (17.4)	0.45 (0.21)	0.26 (0.22)
60–69	53 925 (14.1)	0.47 (0.21)	0.28 (0.23)
70–79	44 370 (11.6)	0.48 (0.21)	0.30 (0.23)
≥80	31 141 (8.2)	0.47 (0.21)	0.30 (0.22)

**Sex**			
Female	217 592 (57.0)	0.43 (0.20)	0.24 (0.21)
Male	163 882 (43.0)	0.47 (0.21)	0.27 (0.23)

**Area deprivation**			
Most deprived^a^	71 416 (18.7)	0.43 (0.20)	0.24 (0.21)
Least deprived	81 362 (21.3)	0.46 (0.21)	0.26 (0.22)

**Ethnicity**			
Bangladeshi	2901 (0.8)	0.41 (0.19)	0.21 (0.19)
Pakistani	7050 (1.8)	0.43 (0.20)	0.24 (0.21)
Indian	10 459 (2.7)	0.46 (0.21)	0.27 (0.23)
Any other Asian background	6407 (1.7)	0.45 (0.21)	0.26 (0.23)
Black African	7978 (2.1)	0.42 (0.20)	0.22 (0.21)
Black Caribbean	5399 (1.4)	0.42 (0.21)	0.24 (0.22)
Any other Black background	2272 (0.6)	0.42 (0.21)	0.22 (0.21)
Chinese	1891 (0.5)	0.45 (0.21)	0.24 (0.22)
Mixed	5183 (1.4)	0.43 (0.20)	0.23 (0.21)
All other ethnic groups	5466 (1.4)	0.44 (0.21)	0.24 (0.22)
White	326 468 (85.6)	0.45 (0.21)	0.25 (0.22)

**Long-term conditions**			
0–1	253 137 (66.4)	0.45 (0.21)	0.24 (0.22)
≥2	128 337 (33.6)	0.45 (0.21)	0.27 (0.21)

**For those with** ≥**2 long-term conditions**			
≥2 physical long-term conditions	91 031 (23.9)	0.45 (0.21)	0.27 (0.22)
≥2 mental long-term conditions	583 (0.2)	0.45 (0.22)	0.28 (0.24)
≥2 physical and mental long-term conditions	36 723 (9.6)	0.44 (0.21)	0.26 (0.21)

a *Patient resident in one of the 20% most deprived areas of England. COC = Bice–Boxerman continuity of care index. SD = standard deviation. UPC = Usual Provider of Care index.*

### Minimally adjusted models

Analysis adjusted for length of follow-up and total number of consultations confirmed a positive association between advancing age and continuity of care captured by the UPC index (Table 2, model 1). Females experienced lower continuity of care than males. Patients living in more deprived areas experienced lower continuity of care than patients living in areas of lower deprivation. Continuity of care increased with each additional long-term condition.

**Table 2.** Association between UPC and demographic characteristics^a^

**Category**	**Model 1^b^ (*n* = 381 474)**		**Model 2,^c^ multiply adjusted (*n* = 381 474)**		**Model 3,^d^ multiply adjusted (*n* = 381 474)**		**Model 4^e^ (*n*= 128 337)**	

	**Coeff**	**(95% CI)**	**Coeff**	**(95% CI)**	**Coeff**	**(95% CI)**	**Coeff**	**(95% CI)**
**Follow-up time in years**	**−0.015**	(−0.016 to −0.014)	**−0.018**	(−0.019 to −0.017)	**−0.018**	(−0.019 to −0.017)	**−0.016**	(−0.018 to −0.015)

**Total consultations**	**−0.001**	(−0.002 to −0.001)	**−0.002**	(−0.002 to −0.002)	**−0.002**	(−0.002 to −0.002)	**−0.001**	(−0.001 to −0.001)

**Age, years (reference 18–29)**								
30–39	**0.007**	(0.005 to 0.009)	**0.007**	(0.005 to 0.009)	**0.007**	(0.005 to 0.009)	**0.010**	(0.004 to 0.016)
40–49	**0.027**	(0.025 to 0.029)	**0.026**	(0.024 to 0.028)	**0.025**	(0.023 to 0.027)	**0.032**	(0.026 to 0.037)
50–59	**0.043**	(0.041 to 0.045)	**0.041**	(0.039 to 0.043)	**0.041**	(0.039 to 0.043)	**0.044**	(0.039 to 0.049)
60–69	**0.059**	(0.057 to 0.061)	**0.056**	(0.054 to 0.058)	**0.056**	(0.054 to 0.058)	**0.060**	(0.055 to 0.065)
70–79	**0.075**	(0.073 to 0.077)	**0.072**	(0.069 to 0.074)	**0.073**	(0.071 to 0.076)	**0.071**	(0.066 to 0.076)
≥80	**0.075**	(0.072 to 0.078)	**0.074**	(0.071 to 0.076)	**0.075**	(0.073 to 0.078)	**0.069**	(0.064 to 0.074)

**Female**	**−0.031**	(−0.033 to −0.029)	**−0.027**	(−0.028 to −0.026)	**−0.027**	(−0.028 to −0.026)	**−0.020**	(−0.022 to −0.018)

**Area deprivation (reference Q1)**								
Q2	**−0.003**	(−0.005 to −0.001)	−0.002	(−0.004 to 0.000)	−0.002	(−0.004 to 0.000)	−0.002	(−0.006 to 0.001)
Q3	**−0.007**	(−0.009 to −0.005)	**−0.003**	(−0.005 to −0.001)	**−0.003**	(−0.005 to −0.001)	−0.003	(−0.007 to 0.000)
Q4	**−0.013**	(−0.015 to −0.011)	**−0.007**	(−0.009 to −0.005)	**−0.007**	(−0.010 to −0.005)	**−0.010**	(−0.014 to −0.006)
Q5 (most deprived)	**−0.018**	(−0.020 to −0.015)	**−0.009**	(−0.011 to −0.007)	**−0.010**	(−0.012 to −0.007)	**−0.010**	(−0.014 to −0.006)

**Ethnicity**								
Bangladeshi	**−0.040**	(−0.047 to −0.032)	**−0.026**	(−0.033 to −0.019)	**−0.025**	(−0.032 to −0.018)	**−0.030**	(−0.045 to −0.015)
Pakistani	**−0.022**	(−0.027 to −0.017)	**−0.010**	(−0.014 to −0.005)	**−0.009**	(−0.014 to −0.004)	**−0.014**	(−0.024 to −0.004)
Indian	0.000	(−0.003 to 0.005)	**0.006**	(0.002 to 0.009)	**0.006**	(0.002 to 0.010)	**0.011**	(0.004 to 0.019)
Any other Asian background	−0.004	(−0.008 to 0.001)	0.004	(−0.001 to 0.008)	0.004	(0.000 to 0.009)	**0.011**	(0.001 to 0.021)
Black African	**−0.024**	(−0.028 to −0.019)	**−0.016**	(−0.020 to −0.012)	**−0.015**	(−0.019 to −0.011)	−0.010	(−0.020 to 0.000)
Black Caribbean	**−0.011**	(−0.016 to −0.006)	**−0.013**	(−0.018 to −0.008)	**−0.012**	(−0.017 to −0.007)	**−0.015**	(−0.024 to −0.006)
Any other Black background	**−0.026**	(−0.033 to −0.018)	**−0.015**	(−0.023 to −0.008)	**−0.015**	(−0.022 to −0.007)	**−0.015**	(−0.033 to −0.003)
Chinese	**0.010**	(0.001 to 0.018)	**0.015**	(0.007 to 0.023)	**0.016**	(0.007 to 0.024)	**0.025**	(0.004 to 0.046)
Mixed	**−0.016**	(−0.021 to −0.011)	−0.004	(−0.009 to 0.001)	−0.004	(−0.009 to 0.001)	0.000	(−0.011 to 0.011)
All other ethnic groups	**−0.014**	(−0.019 to −0.009)	−0.005	(−0.010 to 0.000)	−0.004	(−0.006 to 0.001)	0.009	(−0.003 to 0.021)

**Long-term conditions**								
Each additional long-term condition	**0.007**	(0.007 to 0.008)	**−0.001**	(−0.002 to −0.001)	—	—	—	—
≥2 physical long-term conditions	**0.021**	(0.019 to 0.022)	—	—	**−0.007**	(−0.009 to −0.006)	—	—
≥2 physical and mental long-term conditions	**0.018**	(0.016 to 0.020)	—	—	**0.008**	(0.006 to 0.010)	—	—
≥2 mental long-term conditions	**0.026**	(0.011 to 0.041)	—	—	**0.027**	(0.012 to 0.041)	—	—

a *Bold indicates* P <*0.05.*

b *Model 1: includes follow-up time + number of consultations + GP practice random intercept + each covariate separately.*

c *Model 2: includes follow-up time + number of consultations + GP practice random intercept + age + sex + area deprivation + number of long-term conditions + ethnicity.*

d *Model 3: includes follow-up time + number of consultations + GP practice random intercept + age + sex + area deprivation + combination of long-term conditions + ethnicity.*

e *Model 4: subgroup with* ≥*2 long-term conditions. CI = confidence interval. Coeff = coefficient. Q = quartile. UPC = Usual Provider of Care index.*

### Multiply adjusted model — covariates

Multiply adjusted associations between continuity of care and demographic and clinical covariates were in the same direction as for the bivariate analysis, with the exception of long-term conditions. In the multiply adjusted analysis, continuity of care decreased with each additional long-term condition. Compared with people with ≤1 condition, those with ≥2 physical health conditions had lower continuity of care, but continuity remained higher for those with ≥2 mental health conditions or a combination of physical and mental health conditions.

### Multiply adjusted model — differences across ethnic groups

**Figure 2.**
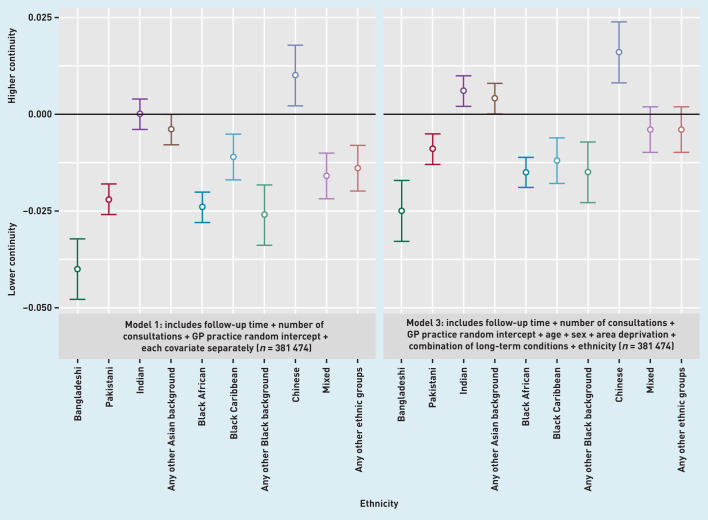
*Difference in continuity of care across ethnic groups. White ethnic group is used as the reference.*

In multiply adjusted analysis, 5 of the 10 ethnic minority groups (Bangladeshi, Pakistani, Black African, Black Caribbean, and any other Black background) had statistically significantly lower continuity of care than patients of White ethnicity (Table 2, models 2 and 3, and Figure 2). Patients of Indian or Chinese ethnicity had higher continuity than patients of White ethnicity. These differences in continuity across ethnic groups remained after adjustment for socioeconomic deprivation in the patient’s local area; being from an ethnic minority group and living in an area with greater deprivation were independently associated with lower continuity.

There was no evidence of an interaction between ethnicity and long-term conditions. The same ethnic differences in UPC were seen for all patients and for the subgroup of patients with ≥2 long-term conditions (Table 2, model 4).

### Sensitivity analyses

The same patterns of ethnic inequalities in continuity were seen for the COC index (Supplementary Table S4).

Analysis was repeated with continuity of care measured over the first 12 months of follow-up from 1 January to 31 December 2016. The subset of patients with ≥3 consultations during this shorter follow-up period were older, more likely to be female, more likely to live in deprived areas, and more likely to have ≥2 long-term conditions compared with the main analytical sample (Supplementary Table S2). Mean UPC (0.57, standard deviation [SD] 0.23) and COC (0.35, SD 0.29) were higher for this subsample, as expected given the shorter follow-up time. For both indices, the associations between continuity of care over 12 months and covariates were of similar magnitude and in the same direction as the main analyses (Supplementary Table S5). Differences in continuity of care over 12 months across ethnic groups were also similar. Statistically significant lower levels of continuity of care were seen for patients from Bangladeshi, Pakistani, Black African, and Black Caribbean ethnic groups compared with patients of White ethnicity.

When the White ethnic group was disaggregated, lower continuity of care was seen for patients in the Irish ethnic group and higher continuity of care in the other White ethnic group compared with the White British ethnic group on both continuity of care indices (Supplementary Table S6). Mixed ethnic groups did not differ significantly from those in the White British ethnic group. As with the main analysis, people of Bangladeshi, Pakistani, Black African, Black Caribbean, and any other Black background ethnic groups had lower continuity of care.

## DISCUSSION

### Summary

Continuity of care was lower for all Black ethnic groups and for Pakistani and Bangladeshi ethnic groups than for the White ethnic group. In the slightly smaller sample where it was possible to disaggregate the White ethnic group, White Irish people are seen to have lower continuity than White British people. Ethnic inequalities in continuity of care persist on adjustment for the number and type of long-term conditions present and for socioeconomic deprivation. Being from an ethnic minority group and living in a socioeconomically deprived area have additive associations with lower continuity of care.

Associations are consistent for different indices of continuity of care and different lengths of follow-up. The differences between ethnic minority groups and the White ethnic group are similar in size to the differences between the most and least deprived fifth of areas. Differences of this size likely have a modest impact on outcomes; previous studies (set outside the UK) suggest that a 0.1 difference in either the UPC^36^ or the COC^37^ index is associated with a modest reduction in mortality risk.

### Strengths and limitations

The study used a large sample of general practice data. With over 95% of the England population registered with a GP,^28^ the sample is nationally representative. Two indices were used in this study to capture different aspects of continuity of care and considered continuity over 1–4 years of follow-up. It was possible to disaggregate ethnicity, although the main analysis used all White ethnic groups as the comparator to minimise loss because of missing detailed ethnicity data. The main analysis may underestimate ethnic inequalities because the White Irish ethnic group had lower continuity than the White British ethnic group.

As with most previous studies, only consultations with GPs were considered. There are increasing numbers of consultations with practice nurses and allied health professionals so it would be of interest to additionally examine ethnic and other differences in continuity in those relationships.

The study controlled for key patient factors, including the presence or absence of >30 long-term conditions and combinations of physical and mental health conditions. However, data on practice factors were not available to the authors. Several practice-level factors, including those relating to the supply of and demand for GP appointments, are modifiable features that can promote or inhibit opportunities to provide continuity of care.

### Comparison with existing literature

Previous studies of patients in England have used cross-sectional survey data to examine preferences for and experience of relational continuity of care across ethnic groups. A study of >2 million responders to the 2009/2010 GPPS described continuity across detailed ethnic groups.^17^ All ethnic minority groups were less likely to see their preferred doctor most of the time compared with those of White British ethnicity. The present findings align with that study for Black, Bangladeshi, and Pakistani groups but not for patients of Indian or Chinese ethnicity. That study adjusted for a similar set of covariates including sex, age group, deprivation quintile, and presence of long-term medical and psychological conditions. They additionally adjusted for number of GPs in the practice and the urgency of appointments the patient had recently sought, but these adjustments did not alter the direction of differences in continuity across ethnic groups.

Several other analyses of surveys of general practice have shown poorer experience of primary health care, including experience of making appointments, communication, interpersonal care, and continuity of care in most ethnic minority groups.^18^^,^^19^^,^^38^ The present study and one other^20^ using routine health data indicate that differences in expectations and other aspects of self-reporting bias^16^^,^^39^ are not feasible explanations for these ethnic inequalities.

### Implications for research and practice

Continuity of care is valued by patients and GPs^40^ but this research shows that it is less available to some ethnic minority groups, including for people with multiple long-term conditions. It is plausible that aspects of healthcare delivery, including lower continuity of care, could contribute to poorer outcomes for people with multiple long-term conditions from ethnic minority groups, although this was not directly tested in the present study. Insufficient local service support to help manage long-term conditions has been described in the lived experience of people from ethnic minority groups.^41^ Given the established associations between continuity of care and adverse outcomes, including higher mortality, unplanned admissions, and complications,^1^^–^^3^ further analysis is required to test and quantify continuity as a possible link between ethnicity and these outcomes. Continuity of care has been identified as one way of tackling inequalities in other medical specialties. For example, the NHS Long Term Plan committed to improving continuity of care during pregnancy as one approach to tackling ethnic inequalities in maternity outcomes.^42^ The findings in the present study suggest that there could be a need to tackle inequalities in continuity of care in primary care, with possible implications for improving outcomes for people from ethnic minority groups with long-term conditions.

The present study did not explore barriers and enablers to continuity of care. There are several practice-level factors that may affect continuity and may result in differences across ethnic groups. These include aspects of demand for general practice, such as the size of the practice (with practices with a larger list size having lower continuity^14^^,^^43^), the number of new registrations, the health of the practice catchment area population, and local socioeconomic deprivation.^16^

Aspects of supply may also be related to continuity. Practices differ in how they balance rapid access to GPs with promoting higher continuity of care. GP staffing patterns, booking systems, and the total number of appointments available to book are also likely to affect patients’ ability to see a preferred GP. Continuity of care is lower where there are more part-time GPs or more GPs in the practice, and is higher in single-handed practices.^17^^,^^44^^,^^45^ There are also national drivers that likely affect access and the ability of practices to offer higher continuity. General practice in deprived areas is underfunded and underdoctored relative to need.^46^ Making the distribution of funding between general practices more equitable, and developing workforce initiatives to attract and retain general practice staff in underdoctored areas^47^ may reduce inequalities in continuity of care by increasing supply.

Several of the factors discussed above — the health of the practice catchment area population, underfunding and underdoctoring relative to need, and area-level socioeconomic deprivation — are unequally distributed across ethnic groups. These are manifestations of structural racism. Structural racism refers to the way in which societies foster racial discrimination through mutually reinforcing systems of health care, housing, education, employment, criminal justice, earnings, and benefits, among others. These patterns and practices in turn reinforce discriminatory beliefs, values, and the distribution of resources.^48^ The structural factors operating across the life course^49^ that lead to overrepresentation of ethnic minority groups in more socioeconomically deprived areas will therefore make a contribution to the inequalities in continuity of care seen in this and other studies. The persistence of the association between ethnicity and continuity even after statistically controlling for area deprivation indicates that additional pathways may be operating. Racism may have additional effects on continuity of care through differences in the way that people from ethnic minority groups are treated in GP practices or experience barriers in living near and accessing the highest level of GP services. Sociocultural norms and language barriers may also contribute to ethnic differences in what patients expect or feel they can influence, and this could affect the extent to which people from some ethnic minority groups seek continuity of care.^50^^,^^51^

Further research is needed to understand why some ethnic minority groups have poorer continuity of care and to identify initiatives that could be made to improve services to meet their needs. The patient–practitioner relationship that is developed through higher continuity of care could help bridge cultural differences and reduce the lack of trust, experiences of insensitive behaviour, and lack of listening that is more commonly experienced in healthcare settings by people from some ethnic minority groups.^51^

Studies show that continuity of care has been declining in England in recent years.^14^^,^^15^ It is not yet clear how the introduction of primary care networks, the chronic shortage of GPs, the increasing use of remote consultations, and other changes in general practice will affect continuity^52^^,^^53^ and inequalities in continuity as the UK recovers from the long-term effects of the pandemic. Ongoing monitoring will be needed.

In conclusion, this analysis of routine health record data from a large sample of patients followed over 4 years shows that relational continuity of care is lower for people from Black African, Black Caribbean, any other Black background, Bangladeshi, and Pakistani ethnic groups. These ethnic inequalities are not accounted for by socioeconomic deprivation and are seen for people with and without multiple long-term conditions.

## Data

This study used data from the CPRD. CPRD data may be accessed by *bona fide* researchers for public health research who are funded by trustworthy organisations. New requests to access the data are assessed through a Research Data Governance process: https://www.cprd.com/Data-access.
